# Anti-inflammatory polyketides from *Santalum album* derived endophytic fungus *Hypomontagnella* sp. TX-09

**DOI:** 10.1080/21501203.2024.2397600

**Published:** 2024-10-16

**Authors:** Xin Ouyang, Senhua Chen, Qiling Chen, Heng Guo, Lan Liu, Hongju Liu, Chong Yan

**Affiliations:** a School of Pharmacy, Guangdong Medical University, Dongguan, China; b School of Marine Sciences, Sun Yat-sen University, Zhuhai, China

**Keywords:** Polyketides, endophytic fungus, *Hypomontagnella* sp., anti-inflammatory activity

## Abstract

Four new lactones, including hypomonacid A (**1**) and hypomonone A–C (**4–6**), as well as nine known polyketide analogues (**2–3** and **7–13**) were obtained from endophytic fungus *Hypomontagnella* sp. TX-09 derived from *Santalum album*. Their planar structures were extensively established by analysing HRESIMS and NMR spectroscopic data. Stereochemistry of new compounds was determined by X-ray diffraction analysis and modified Mosher’s method in combination with quantum-chemical ECD calculation. In addition, compounds **1** and **2** showed anti-inflammatory activity by inhibition of lipopolysaccharide (LPS)-induced NO production in RAW264.7 cells at 50 μmol/L without cytotoxicity. Among them, compound **1** inhibited the production of LPS-stimulated inflammation in mouse macrophage RAW264.7 cells by suppressing the expression of iNOS, TNF-α, IL-1β, and IL-6.

## 1. Introduction

Symbiotic microorganisms known as plant endophytic fungi live inside the tissues of their host plants for all or part of their life cycles without causing apparent disease symptoms (Bhunjun et al. 2024). These hosts provide essential nutrients and a protective environment for the endophytes (Khare et al. 2018). In return, endophytes produce a diverse array of bioactive secondary metabolites that enhance the growth and ecological competitiveness of their host plants (Srinivasa et al. 2022). Numerous natural compounds with a variety of chemical structures and biological activity can be produced by endophytic fungi (Shi et al. 2024). These substances can be classified as alkaloids, steroids, terpenoids, glycosides, and polyketides. On Earth, there are about 300,000 higher plant species, each potentially hosting one or more endophytic fungi (Nisa et al. 2015). However, only a small number of these symbiotic relationships have been thoroughly explored (Adnani et al. 2017). In recent years, the use of endophytic fungi’s beneficial secondary metabolites has garnered attention (Gupta et al. 2020). Some of these secondary metabolites are lead compounds with potential as clinical drug candidates for anti-cancer, antiviral, anti-inflammatory, antibacterial, and antifungal activities (Chen et al. 2022).

**Figure 1.**
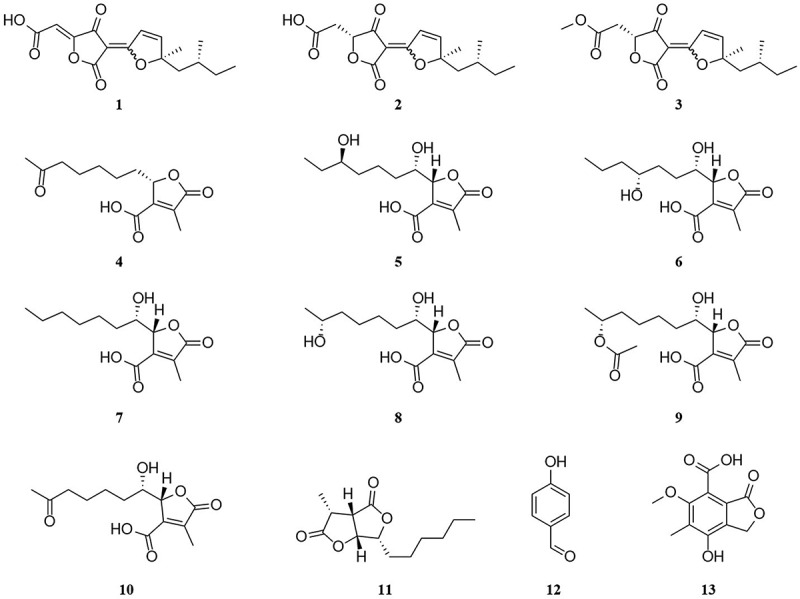
Chemical structures of compounds **1–13**.

The plant endophytic fungus *Hypomontagnella* sp. TX-09 was recently isolated from the Indian sandalwood’s heartwood. Its fermentation broth’s EtOAc extract showed modest anti-inflammatory effects. Thirteen polyketides were isolated from the crude extract after further chemical analysis. These included nine known polyketides and four novel lactones, including hypomonacid A (**1**) and hypomonone A–C (**4–6**) (Figure 1). 1D and 2D NMR, modified Mosher’s technique, X-ray diffraction analysis, and ECD calculation were adopted to fix the chemical structures of the isolates. Compounds **1** and **2** have an inhibitory effect on suppression of lipopolysaccharide-induced nitric oxide production in RAW264.7 cells.

## 2. Materials and methods

### 2.1. General experimental procedures

The optical rotation values were measured with an MCP 200 polarimeter. Infrared radiation (IR) spectra were measured by an infrared spectrometer (EQUINOX 55, Bruker). The ultraviolet (UV) spectra were obtained on a Blue Star spectrophotometer. The ECD spectra were recorded using the Chirascan CD Spectrometer. All NMR spectra were measured on Bruker Avance nuclear magnetic resonance spectrometer (400 or 600 MHz). A Thermo Corporation LTQ-Orbitrap LC-MS spectrometer was used to record HRESIMS data. Sephadex LH-20 and silica gel (200–300 mesh) were used for compound’s purification performed on column chromatography. Further purification was carried out by High Performance Liquid Chromatography.

### 2.2. Fungal material

The heartwood section of Indian sandalwood was collected in July 2021. From the heartwood, a strain designated as TX-09 was identified and conserved at Marine Natural Products Research Group, Sun Yat-Sen University. The fungal genus was identified as *Hypomontagnella* sp. based on molecular data (ITS region) using a BLAST search in GenBank (Raja et al. 2017). The strain’s ITS sequence data has been submitted to the GenBank database (accession number: PP716768).

### 2.3. Extraction and isolation

The strain *Hypomontagnella* sp. TX-09 was grown in 134 Erlenmeyer flasks of sold-rice medium containing 35 g rice and 45 mL distilled water at room temperature for 30 days. Following fermentation, the culture was soaked in methanol solvent with three times and thoroughly concentrated, and the dry ethyl acetate crude extract (87 g) was provided by extracting the culture sequentially with H_2_O and EtOAc. Then, ethyl acetate extract was performed on column chromatography using 200–300 mesh silica gel, and seven fractions (A–G) were obtained by eluting petroleum ether and ethyl acetate solvent from 100:0 to 0:100 with increasing polarity and methanol solvent. Using a Sephadex LH-20 column, fraction B was processed, producing compound **11** (67 mg). Three subfractions (C-1 to C-3) were obtained from fraction C after it was run through a Sephadex LH-20 column. Using an Agilent-SB-C18 column operating at 3 mL/min and 35% MeCN/H_2_O, subfraction C-2 was applied to RP-HPLC, producing compounds **12** (3.2 mg) and **13** (3.0 mg). Fraction D was subjected to a Sephadex LH-20 column chromatography eluting with CH_2_Cl_2_/MeOH (1:1), resulting in three subfractions (D-1 to D-3). Subfraction D-1 was purified by silica gel column chromatography using CH_2_Cl_2_/MeOH (50:1) solvent. Then, RP-HPLC with MeCN/H_2_O (30:70, 3 mL/min) yielded three compounds, including **1** (104 mg), **2** (1,261 mg), and **3** (86 mg). RP-HPLC (MeCN/H_2_O = 35:65, 3 mL/min) was used to further purify subfraction D-2. The results showed that compounds **6** (3.4 mg), **7** (643.1 mg), **8** (877.05 mg), and **9** (15.9 mg). Using RP-HPLC (MeCN/H_2_O = 25:75, 3 mL/min), subfraction D-3 was further purified, resulting in compounds **4** (44 mg), **5** (77 mg), and **10** (4.7 mg).

Hypomonacid A (**1**): yellow crystal; [*α*]$\begin{array}{c} 25\\ D \end{array}$ 250 (MeOH, *c* 0.1); UV (MeOH) *λ*_max_ (log *ε*) 334.8 (4.05) nm; IR (neat) *ν*_max_ 3,475, 3,361, 2,956, 2,921, 2,850, 1,741, 1,662, 1,631, 1,361, 1,259, 1,111, 972 cm^−1^; ^1^H NMR (400 MHz) and^13^C NMR (100 MHz) data was shown in Table 1; HR-ESIMS *m/z* 307.1176 [M+H]^+^ (calcd for C_16_H_19_O_6_, 307.1176).

**Table 1.** ^1^H and^13^C NMR spectroscopic data of compound **1** in CDCl_3._

	1				
Position	*δ*_C_		*δ*_H_, mutl (*J* in Hz)		Type
	*E*	*Z*	*E*	*Z*	
1	168.4	168.5			C
2	92.0	91.5			C
3	180.1	180.9			C
4	154.3	153.9			C
5	95.3	95.2	5.89, d (3.9)	5.88, d (3.9)	CH
6	166.1	166.1			C
1’	181.9	182.2			C
2’	123.0	122.8	7.56, d (5.7)	7.41, d (5.7)	CH
3’	163.3	163.2	7.67, d (3.5)	7.65, d (4.0)	CH
4’	103.9	103.3			C
5’	44.4	44.4	2.13, d (3.3)	2.08, d (3.5)	CH_2_
6’	30.3	30.3	1.27, m	1.25, m	CH
7’	30.4	30.4	1.17, m	1.16, m	CH_2_
8’	11.1	11.1	0.82, t (7.2)	0.8, t (7.2)	CH_3_
9’	23.6	23.6	1.63, s	1.63, s	CH_3_
10’	20.9	20.8	0.89, d (4.9)	0.89, d (5.0)	CH_3_

Hypomonone A (**4**): yellow oil; [*α*]$\begin{array}{c} 25\\ D \end{array}$ −28.5 (MeOH, *c* 0.1); UV (MeOH) *λ*_max_ (log *ε*) 228 (3.86) nm; ECD (MeOH) *λ*_max_ (Δ*ε*) 226.7 (3.9) nm; IR (neat) *ν*_max_ 3,347.2, 1,658.7, 1,017.6 cm^−1^; ^1^H NMR (400 MHz) and^13^C NMR (100 MHz) data was shown in Table 2; HR-ESIMS *m/z* 255.1230 [M+H]^+^ (calcd for C_13_H_19_O_5_, 255.1227).

**Table 2.** ^1^H and^13^C NMR spectroscopic data of compounds (**4** and **5** in MeOD, **6** in CDCl_3_).

Position	4		5		6	
	*δ*_C_, type	*δ*_H_ (*J* in Hz)	*δ*_C_, type	*δ*_H_ (*J* in Hz)	*δ*_C_, type	*δ*_H_ (*J* in Hz)
1	173.0, C		175.5, C		175.5, C	
2	138.3, C		138.3, C		138.1, C	
3	147.5, C		148.4, C		148.6, C	
4	81.4, CH	5.12, m	85.3, CH	4.99, s	85.4, CH	5.11, s
5	32.5, CH_2_	2.11, m	70.3, CH	4.06, dd (8.0, 5.1)	71.7, CH	4.10, dd (9.4, 4.8)
6	24.6, CH_2_	1.42, m	35.2, CH_2_	1.58, m	31.4, CH_2_	1.76, m
7	28.7, CH_2_	1.32, m	23.3, CH_2_	1.44, m	34.7, CH_2_	1.51, m
8	23.5, CH_2_	1.57, m	37.5, CH_2_	1.36, m	70.4, CH	3.50, m
9	43.6, CH_2_	2.43, t (7.3)	73.7, CH	3.36, m	40.6, CH_2_	1.36, m
10	209.9, C		31.0, CH_2_	1.37, m	19.9, CH_2_	1.36, m
11	30.1, CH_3_	2.14, s	10.4, CH_3_	0.82, t (7.4)	14.5, CH_3_	0.86, t (0.86)
12	11.1, CH_3_	2.22, s	10.8, CH_3_	2.04, s	10.7, CH_3_	2.08, s
13	164.9, C		164.9, C		165.1, C	

Hypomonone B (**5**): yellow oil; [*α*]$\begin{array}{c} 25\\ D \end{array}$ −81.9 (MeOH, *c* 0.1); UV (MeOH) *λ*_max_ (log *ε*) 229 (3.94) nm; ECD (MeOH) *λ*_max_ (Δ*ε*) 207.2 (−9.8), 247.1 (2.8) nm; IR (neat) *ν*_max_ 3,384.4, 2,929.7, 1,740.7, 1,226.3 cm^−1^; ^1^H NMR (400 MHz) and^13^C NMR (100 MHz) data was shown in Table 2; HR-ESIMS *m/z* 271.1177 [M-H]^−^ (calcd for C_13_H_19_O_6_, 271.1182).

Hypomonone C (**6**): yellow oil; [*α*]$\begin{array}{c} 25\\ D \end{array}$ −130.0 (MeOH, *c* 0.1); UV (MeOH) *λ*_max_ (log *ε*) 228 (4.03) nm; ECD (MeOH) *λ*_max_ (Δ*ε*) 207.2 (−8.7), 247.1 (2.4) nm; IR (neat) *ν*_max_ 3,347.2, 1,658.7, 1,017.6 cm^−1^; ^1^H NMR (400 MHz) and^13^C NMR (100 MHz) data was shown in Table 2; HR-ESIMS *m/z* 271.1179 [M-H]^−^ (calcd for C_13_H_19_O_6_, 271.1182).

### 2.4. Modified Mosher’s method

The experiment to ascertain the secondary hydroxyl group structure of compounds **5–6** was conducted using a modified version of Mosher’s approach (Guo et al. 2021; Jiang et al. 2023). In short, 0.8–1 mg of the sample was dissolved in deuterated pyridine solvent (0.5 mL) with an NMR tube after being dried for 24 h in a vacuum dryer. Next, (*S*)-MTPA ester (**a**) was obtained by adding 5.0 µL (*R*)-(-)-MTPA chloride to the NMR tube at room temperature. The same sample was used again, but instead of the pretreatment described above, the reaction was supplemented with 5.0 µL of (*S*)-(+)-MTPA chloride to give (*R*)-MTPA ester (**b**). Twenty minutes later, each reaction product was recorded on^1^H NMR (400 MHz, pyridine-*d*_5_) and^1^H-^1^H COSY spectra. Finally, the configuration of secondary hydroxyl group could be assigned by comparing their NMR data.

(*S*)-MTPA Ester of **5** (**5a**): ^1^H NMR *δ*_H_: 5.0647 (2 H, m, H-5/9), 1.5703 (2 H, m, H-10), 1.4837 (1 H, m, H-8a), 1.0291 (3 H, t, H-11), 0.7049 (1 H, t, H-8b). (*R*)-MTPA Ester of **5** (**5b**): ^1^H NMR *δ*_H_: 5.0911 (1 H, m, H-9), 1.5359 (3 H, m, H-10/8a), 0.8322 (3 H, t, H-11), 0.7159 (1 H, t, H-8b). (*S*)-MTPA Ester of **6** (**6a**): ^1^H NMR *δ*_H_: 5.2242 (1 H, m, H-8), 1.8405 (2 H, m, H-7), 1.4918 (1 H, m, H-9a), 1.4117 (1 H, m, H-9b), 1.1492 (2 H, m, H-10), 0.7177 (3 H, t, H-11). (*R*)-MTPA Ester of **6** (**6b**): ^1^H NMR *δ*_H_: 5.2305 (1 H, m, H-8), 1.7959 (2 H, m, H-7), 1.5681 (1 H, m, H-9a), 1.4271 (1 H, m, H-9b), 1.2616 (2 H, m, H-10), 0.7775 (3 H, t, H-11).

### 2.5. Computational methods

The software package Spartan’14 was used to perform Merck molecular force field (MMFF) calculations from Wavefunction Inc. Additionally, the Gaussian 09 program was used to calculate DFT/TD-DFT (Frisch et al. 2010). Low-energy conformers were found using an MMFF conformational search of less than 10 kcal/mol. The DFT approach [B3LYP/6-31 G(d) level] was then used to optimise these conformers. At the same level, frequency calculations were subjected to quantify the relative thermal free energies (ΔG) of each optimised conformer at 298.15 K and validate their real minimal nature. The low-energy conformers in solvent were determined to have energies at the M06-2X/def2-TZVP level (Zhao and Truhlar 2008). Using Shermo, the Boltzmann distribution, and Gibbs free energy determined the population of each conformer (Lu and Chen 2021). All free energies underwent a free energy correction to take into consideration the change from the gas phase (1 atm) to the liquid phase (1 M). For additional computations, the conformer distribution greater than 3% was selected. The PCM, ECD calculations [PBE1PBE/TZVP or B3LYP/6-311 G (d,p) level] were performed. The experimental CD spectra and calculated curves were compared using the program SpecDis (Bruhn et al. 2013).

### 2.6. X-ray crystal structure analysis

Vapour diffusion was used to produce the colourless crystals of compound **1** (Jiang et al. 2023). The single crystal X-ray diffraction data with Cu-Kα radiation (λ = 1.54178 A) were obtained using a Rigaku Oxford Diffraction system. The structures were solved directly (SHELXS-2014) (Dolomanov et al. 2009), and Olex2 software was used to improve the solutions. Cif data of **1** have been deposited with the number (CCDC: 2350692) at the Cambridge Crystallographic Data Centre.

### 2.7. Cell viability and NO assay

RAW264.7 cells used in the experiment were purchased from the Chinese Academy of Sciences in Shanghai. Every tested compound was made as DMSO stock solutions. RAW264.7 cells were planted in 96-well plates with a density of 5 × 10^4^ cells per well and exposed to different doses of the chemicals and LPS (1 μg/mL) for a duration of 24 h, following the fusion degree reaching 80%. The Cell Counting Kit-8 (CCK-8) was adopted to test the vitality of the cells. Each well received 10 μL of CCK-8 working solution (ZETA life, USA), and the cells were grown for an additional 30 min. The absorbance was then determined using Thermo Scientific’s Multiskan GO at 450 nm. For a duration of 24 h, 96-well plates were seeded with 1 × 10^5^ cells per well of RAW264.7 cells. The cell culture supernatant (50 μL) was pipetted to a fresh 96-well plate 24 h following the dosing procedure. After adding nitric oxide detection reagents I and II, each containing 50 μL per well, the resulting colour was measured at 540 nm.

### 2.8. Real-time PCR

A Trizol RNA extraction kit was used for the extraction of Total RNA. Then, the real-time fluorescence quantitative reverse-transcription kit was used for reverse transcription RNA into cDNA. An initial pre-denaturation step was conducted at 95 °C for 30 s in the PCR reaction programme. This was followed by 45 cycles of denaturation at 95 °C for 5 s each, and a combined extension/annealing step at 60 °C for 1 min. The 2^-ΔΔCt^ technique was utilised to ascertain the relative expression level of the target gene, and the β-actin served as the internal reference gene. The fluorescence intensity of the TB Green I DNA dye was automatically recorded by the PCR apparatus in every cycle. The following primers: β-actin, tumour necrosis factor-α (TNF-α), interleukin-6 (IL-6), and IL-1β were used for PCR analysis as described previously (Chen et al. 2022).

### 2.9. Western blot

Western blotting was used in accordance with conventional protocols to evaluate protein expression. The following antibodies are needed for western blot analysis: β-actin (13E5), cyclooxygenase-2 (COX-2) (ab179800), NO synthase (iNOS) (ab178945), and rabbit IgG H&L (ab6721). The Molecular Imager ChemiDoc XRS+ System was used to detect signals.

### 2.10. Statistical analysis

The Newman-Keuls test was used for multiple comparisons after a one-way ANOVA. GraphPad statistical analysis was performed using Prism 8.0 software. *p* < 0.05 is designated as the significant level, and the data (three independent experiments) are displayed as the mean ± SD.

## 3. Results and discussion

Chemical investigation of EtOAc extracts of the strain *Hypomontagnella* sp. TX-09 led to the purification of 13 polyketides (**1–13**), including four new lactones (**1**, **4–6**) (Figure 1). The eight known polyketides were identified as nodulisporacid (**2**) (Huang et al. 2023), nodulisporacid A methyl ester (**3**) (Huang et al. 2023), isosporothric acid or colisiderin A **(7**) (Surup et al. 2014; Hu et al. 2015), xylariacinic B (**8**) (Yuan et al. 2021), xylariacinic C (**9**) (Yuan et al. 2021), xylariacinic A (**10**) (Yuan et al. 2021), dihydrosporothriolide (**11**) (Surup et al. 2014), *p*-hydroxybenzaldehyde (**12**) (Gao et al. 2023), and 7-carboxy-4-hydroxy-6-methoxy-5-methylphthalide (**13**) (Chen et al. 2021), respectively.

### 3.1. Structural characterization

**Figure 2.**
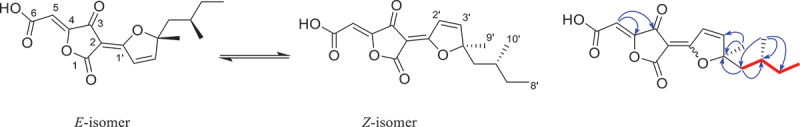
The structure and HMBC (arrows) and^1^H–^1^H COSY (bolds) correlations of compound **1**.

**Figure 3.**
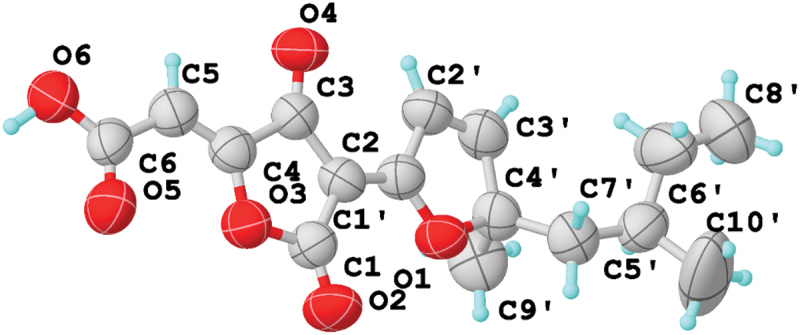
X-ray crystallographic analysis of compound **1**.

Compound **1** has a molecular formula of C_16_H_18_O_6_ on the base of its HRESIMS molecular ion peak *m/z* 307.1179 [M+H]^+^ (calcd for C_16_H_19_O_6_, 307.1176). The *E*- and *Z*-isomers spontaneously interconverted, ultimately equilibrating at a 1:1 *E*/*Z* ratio, according to the ^1^H and ^13^C NMR spectra of **1**. This phenomenon was similar to that of a known compound, nodulisporacid A (**2**) (Kasettrathat et al. 2008), which was also isolated from the strain. The main difference between them was that compound **1** had the presence of an additional double bond and the absence of methylene and methine. This evidence suggested that **1** was dehydrogenation derived from **2**. The double bond’s placement in compound **1** was determined by the key HMBC cross-peaks from olefinic protons H-5 to C-3, C-4, and C-6 (Figure 2). The attempt to use semi-preparative reversed-phase HPLC to separate an equilibrium *E*/*Z* mixture of **1** was unsuccessful. However, using slow evaporation of methanol solvent, *E*/*Z* mixture of **1** was attempted to crystallise, and a yellow crystal was obtained and subsequently analysed by X-ray crystallography (Figure 3). Following the final refinement of the Cu Kα data, it was determined that only the *Z*-isomer was present in the crystals of **1**, leading to the determination of the *Z*-isomer’s chemical structure, which is seen in Figure 3. Therefore, compound **1** was named hypomonacid A.

**Figure 4.**
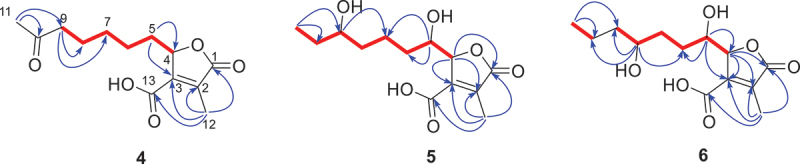
The HMBC (arrows) and^1^H–^1^H COSY (bolds) correlations of **4–6**.

Compound **4** was obtained as yellow oil. The molecular formula of **4** was assigned as C_13_H_18_O_5_ by the positive mode HRESIMS *m/z* 255.1230 [M+H]^+^, confirming the existence of five degrees of unsaturation. Carbonyl group was found via absorption resonance in the IR spectra at 1,658 cm^−1^. The^1^H and^13^C NMR (Table 2) revealed that compound **4** had 13 carbons, including two methyl groups (*δ*_H_ 2.14, *δ*_C_ 30.1; *δ*_H_ 2.22, *δ*_C_ 11.1), five methylene groups (*δ*_H_ 2.11, *δ*_C_ 32.5; *δ*_H_ 1.43, *δ*_C_ 24.6; *δ*_H_ 1.32, *δ*_C_ 28.7; *δ*_H_ 1.57, *δ*_C_ 23.5; *δ*_H_ 2.43, *δ*_C_ 43.6), one oxygenised mentine (*δ*_H_ 5.12, *δ*_C_ 81.4), two olefinic carbon (*δ*_C_ 138.3, 147.5), and three carbonyl groups (*δ*_C_ 173.0, 164.9, 209.9).

**Figure 5.**
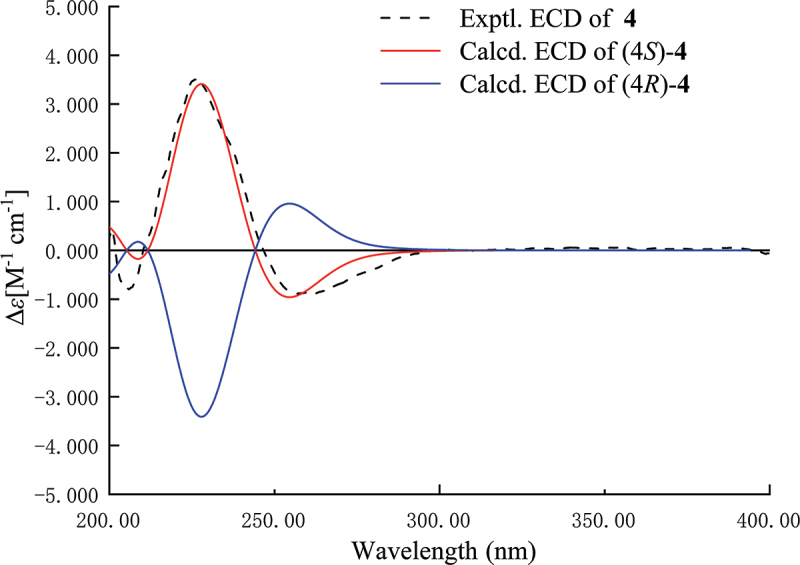
Experimental and calculated ECD spectra of **4** in MeOH solvent.

Analysis of the comprehensive 2D NMR spectroscopic data (Figure 4) elucidated the chemical planar structure of compound **4**. Butyrolactone containing the methyl and carboxylic acid group was determined by the HMBC cross-peaks between H-12 and C-1, C-2, C-3, and C-13. The^1^H–^1^H COSY correlations of H-5/H-6/H-7/H-8/H-9 and key HMBC cross-peaks from H-11 and H-9 to C-10 and were used to build the heptanone group. The HMBC cross-peaks from H-5 to C-3 and C-4 led to the attachment of the heptanone side chain to C-4 of the butyrolactone. The expected ECD spectrum of (*S*)-**4** fitted perfectly with that of the experimental one, suggesting that the absolute configurations of C-**4** should be *S* (Figure 5). Consequently, compound **4** was designated as hypomonone A, a new lactone.

**Figure 6.**
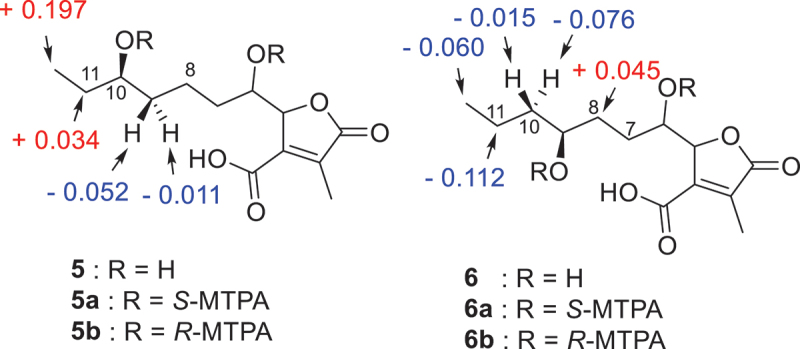
∆*δ* = *δ*_*S*_ − *δ*_*R*_ values (ppm) were acquired from the MTPA esters of compounds **5** and **6**.

**Figure 7.**
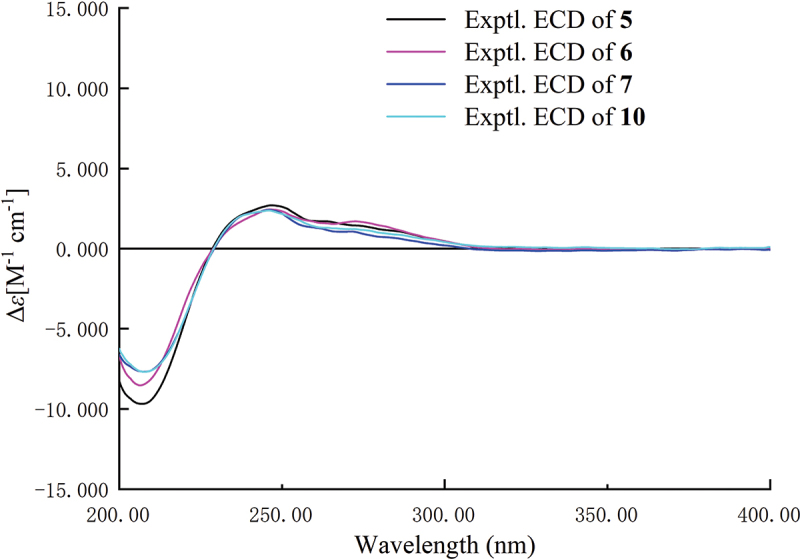
Experimental ECD spectra of compounds **5–7** and **10** (in MeOH).

Compound **5** was obtained as yellow oil. The HRESIMS of **5** displayed a molecular ion peak at *m*/*z* 271.1177 [M-H]^−^ (calcd for C_13_H_19_O_6_, 271.1182), indicating the molecular formula was C_13_H_20_O_6_ and the degrees of unsaturation was four. A butyrolactone core skeleton was also suggested to be present in compound **5** by analysis of the^1^H and^13^C NMR data (Table 2). The molecular structure of **5** had high degree of similarity to that of xylariacinic B (**8**), which was also isolated from the strain. The chemical shift variation of side chain carbons and the peak type variation of terminal methyl protons were observed between them. Peak pattern of terminal methyl protons was changed from double peaks in compound **8** to triple peaks in compound **5**, indicating that the substitution position of the hydroxyl group changed from C-10 to C-9 in **5**. The hydroxyl position was further determined by key HMBC cross-peaks from methyl protons H-11 to oxygenised methine C-9 and methylene C-10. Chemical shift difference (∆*δ* = *δ*_S_ − *δ*_R_) of the proton signals around C-9 were displayed in Figure 6, which was obtained by applying a modified version of Mosher’s approach to determine the chirality of C-9 as *R* in **5**. Compounds **5–7** and **10** exhibited very similar ECD spectra, which have a negative Cotton effect (CE) at 207 nm and a positive CE at 247 nm (Figure 7). Among them, the absolute configurations of colisiderin A (**7**) have been determined by TDDFT-ECD calculation protocol (Hu et al. 2015), which suggested that compounds **5–7** and **10** have the same chirality of C-4 and C-5 with 4*R*, 5*S*. Thus, **5** was also assigned as a new butyrolactone polyketide that was named hypomonone B.

Compound **6** was obtained as yellow oil and given the molecular formula C_13_H_20_O_6_ according to the negative mode *m/z* 271.1179 [M-H]^−^, which has the same molecular formula as that of **5**. Compounds **5** and **6** were found to be a pair of isomers after the analysis of their 1D NMR data (Table 2). The HMBC correlations from H-8 and H-11 to C-9 and C-10 supported the change in substitution position of the hydroxyl group from C-9 in **5** to C-8 in **6**. The Modified Mosher’s method was adopted to identify the chirality of C-8 in **6** as *R* (Figure 6). In the light of biosynthetic considerations and quite similar ECD spectra (Figure 7), the configurations of C-4 and C-5 of **6** were identical to those of **5**, **7–10** with 4*R*, and 5*S*. As a result, **6** was also identified as a new butyrolactone polyketide and named hypomonone C.

**Figure 8.**
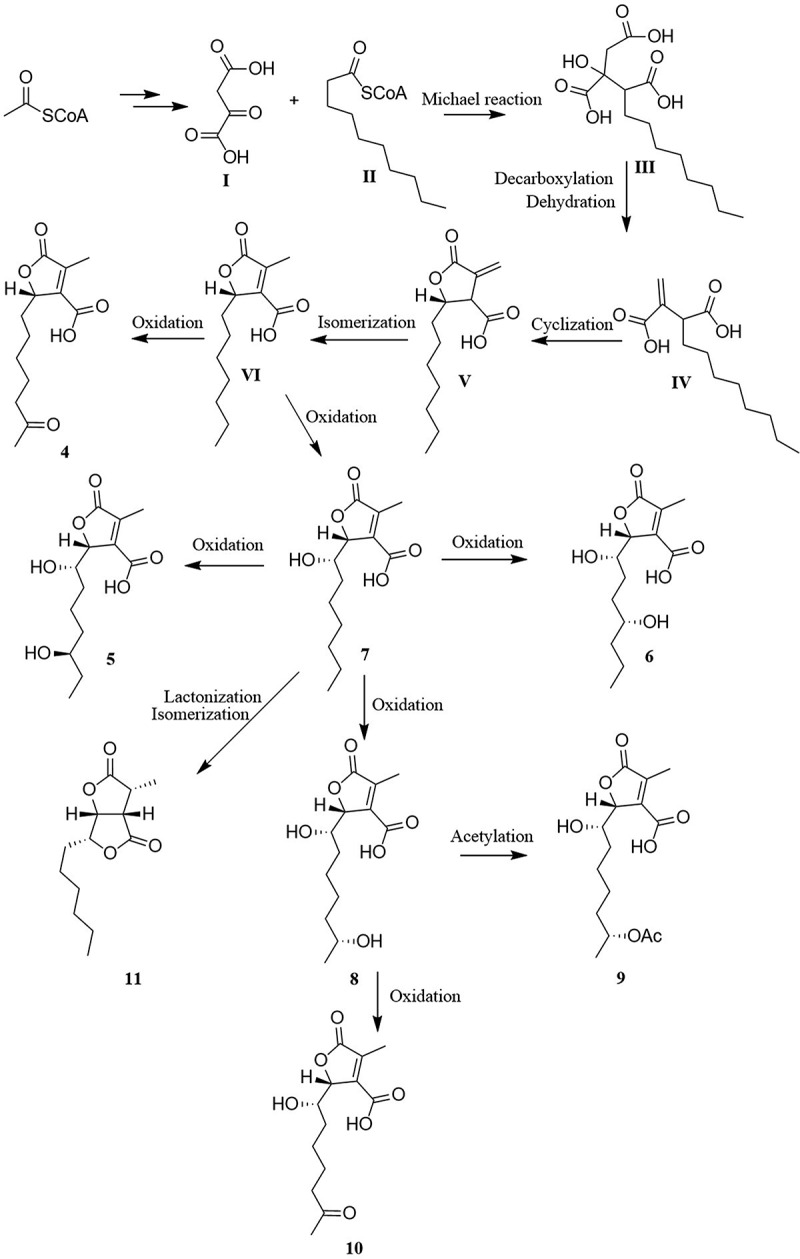
Proposed biosynthetic pathway of compounds **4–11**.

The biosynthesis of compounds **4–11** was proposed to be a PKS biosynthetic pathway (Figure 8). The precursor molecules **I** and **II** could be produced from malonic acid through PKS biosynthetic pathway (Saetang et al. 2020). Then, the intermediate **III** was derived from intermediates **I** and **II** occurring by the Michael reaction. The decarboxylation and dehydration occurring in intermediate **III** constructs an intermediate **IV**. Intermediate **VI** was formed from **IV** followed by cyclisation and isomerisation. Intermediate **VI** converted to compounds **4** and **7** by oxidation and hydroxylation, respectively. Alternatively, compounds **6**, **5**, and **8** were obtained from compound **7** undergoing hydroxylation at C-8, C-9, and C-10. Compound **8** undergoes oxidation and acetylation to yield compounds **10** and **9**, respectively. Lactonization and isomerisation of compound **7** would generate compound **11**. Due to the same PKS biosynthetic pathway from intermediate **VI**, it is worth noting that C-4 of **4–10** and C-5 of **5–10** possess identical configurations 4*R* and 5*S*, respectively.

### 3.2. Anti-inflammatory activity

**Figure 9.**
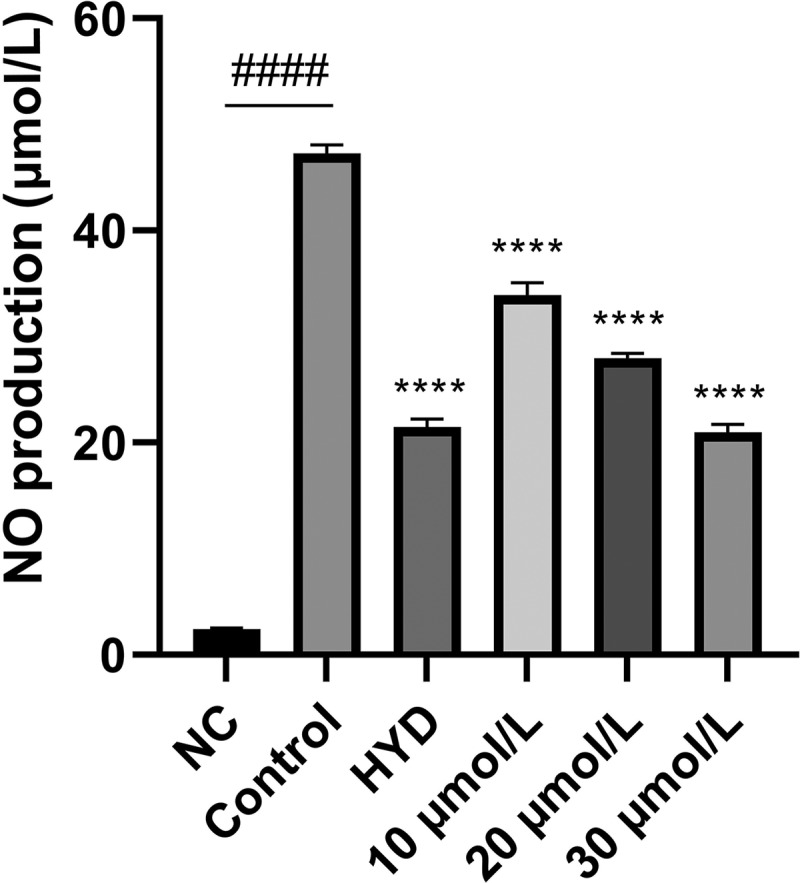
Effects of lps-induced nitric oxide production by compound **1** in RAW 264.7 cells. ****p < 0.0001 vs control group, and ^####^*p* < 0.0001 vs negative control (NC) group.

Compounds **1–13** were tested for their anti-inflammatory properties in RAW264.7 cells stimulated with LPS, and Hydrocortisone (HYD) was used as a positive control (Zhang et al. 2022). Initially, we observed that compounds **1–13** inhibited the production of NO, a critical inflammatory mediator, stimulated by LPS in RAW 264.7 cells. Compounds **1−3** significantly inhibited NO production at 50 μmol/L by 76.06%, 53.55%, and 88.48%, respectively. To rule out the possibility that cell toxicity was to blame for the compounds’ inhibition of NO production, the cytotoxicity of the compounds was assayed by CCK-8. It was found that compounds **1** and **2** did not exhibit cytotoxicity at 50 μmol/L, whereas compound **3** was highly cytotoxic with cell survival rate of 1.90%. This indicates that compounds **1** and **2** have NO inhibitory activity. Further study showed that compound **1** had dose-dependent inhibition of NO production at 10, 20, and 30 μmol/L (Figure 9).

**Figure 10.**
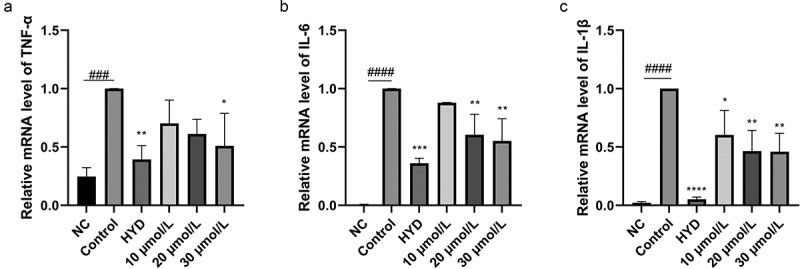
Inhibition of lps-induced pro-inflammatory cytokines TNF-α (a), IL-6 (b), and IL-1β (c) by compound **1** at the mRNA level in RAW 264.7 cells. *p < 0. 05, **p < 0. 01, ***p < 0.001 and ****p < 0.0001 vs control group, ###*p* < 0.001 and ####*p* < 0.0001 vs negative control (NC) group.

**Figure 11.**
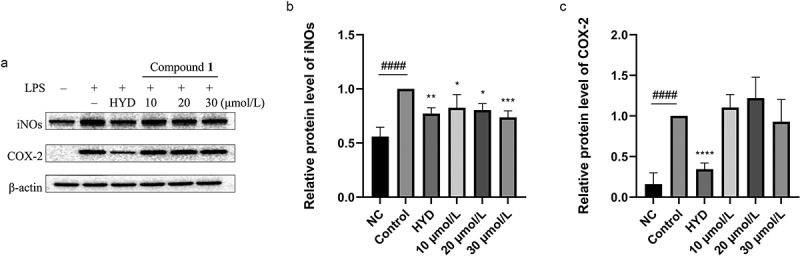
Effect of compound **1** on iNOS and COX-2 protein expression in RAW 264.7 cells. Representative protein bands from Western blot (a). Quantitative analysis of iNOS (b) and COX-2 (c) Western blot proteins bands. *p < 0. 05, **p < 0. 01, ***p < 0.001 and ****p < 0.0001 vs control group, ###*p* < 0.001 and ####*p* < 0.0001 vs negative control (NC) group.

LPS can activate macrophages through cell-surface-expressed Toll-like receptors (TLRs) to induce inflammation and produce pro-inflammatory cytokines, including TNF-α, IL-1β, and IL-6, and inflammatory mediators, such as NO and prostaglandin E2 (PGE2), which are created by iNOS and COX-2, respectively (Wang et al. 2016). The result showed that compound **1** effectively suppressed the mRNA expression of inflammatory factors TNF-α, IL-6, and IL-1β (Figure 10a). Also compound **1** greatly decreased the expression level of iNOS protein (Figure 11b), which was consistent with NO assay results. But compound **1** had no effect on COX-2 (Figure 11c). Above results suggested that **1** demonstrated the ability of anti-inflammation.

## 4. Conclusions

To summarise, we have discovered four previously unidentified lactones (**1**, **4−6**) and nine known biogenetic derivatives (**2−3** and **7−13**) from the endophytic fungus *Hypomontagnella* sp. TX-09 that was derived from *Santalum album*. The stereochemistry of new compounds was elucidated in combination with ECD calculations, X-ray diffraction, and modified Mosher’s method. Anti-inflammatory pharmacological studies revealed that **1** and **2** have a strong anti-inflammatory effect against LPS-induced inflammation.

## Supplementary Material

final_Supporting_Information.docx
