# Supplementary material for: Anti-inflammatory polyketides from Santalum album derived endophytic fungus Hypomontagnella sp. TX-09
Source: Mycology. 2024 Oct 16;16(2):918–28. doi: 10.1080/21501203.2024.2397600 (PMC12096673; doi:10.1080/21501203.2024.2397600)
Supplement: final_Supporting_Information.docx [file TMYC_A_2397600_SM1210.docx]

**Anti-Inflammatory polyketides from *Santalum album* derived endophytic fungus *Hypomontagnella* sp. TX-09**

Xin Ouyang^a, #^, Senhua Chen^b, #^, Qiling Chen^a^, Heng Guo^b^, Lan Liu^b,c^, Hongju Liu^a,*^, Chong Yan^a,*^

^a^School of Pharmacy, Guangdong Medical University, Dongguan, 523808, China.

^b^School of Marine Sciences, Sun Yat-sen University, Zhuhai 519000, China.

^*^Corresponding authors:

Chong Yan jdsbj2000@163.com

Hongju Liu lhju88@126.com

School of Pharmacy, Guangdong Medical University, Dongguan, 523808, China

^#^These authors contributed equally to this work

[Figure S1. The HR-ESIMS of compound **1**. 4](#_Toc168325069)

[Figure S2. The ^1^H NMR (400 MHz) spectrum of compound **1** in CDCl_3_. 4](#_Toc168325070)

[Figure S3. The ^13^C NMR (100 MHz) spectrum of compound **1** in CDCl_3_. 5](#_Toc168325071)

[Figure S4. The HSQC spectrum of compound **1** in CDCl_3_. 5](#_Toc168325072)

[Figure S5. The ^1^H-^1^H COSY spectrum of compound **1** in CDCl_3_. 6](#_Toc168325073)

[Figure S6. The HMBC spectrum of compound **1** in CDCl_3_. 6](#_Toc168325074)

[Figure S7. The NOESY spectrum of compound **1** in CDCl_3_. 7](#_Toc168325075)

[Figure S8. The IR spectrum of compound **1**. 7](#_Toc168325076)

[Figure S9. The. UV spectrum of compound **1**. 8](#_Toc168325077)

[Figure S10. The HR-ESIMS of compound **4** . 8](#_Toc168325078)

[Figure S11. The ^1^H NMR (400 MHz) spectrum of compound **4** in CDCl_3_. 9](#_Toc168325079)

[Figure S12. The ^13^C NMR (100 MHz) spectrum of compound **4** in CDCl_3_. 9](#_Toc168325080)

[Figure S13. The HSQC spectrum of compound **4** in CDCl_3_. 10](#_Toc168325081)

[Figure S14. The ^1^H-^1^H COSY spectrum of compound **4** in CDCl_3_. 10](#_Toc168325082)

[Figure S15. The HMBC spectrum of compound **4** in CDCl_3_. 11](#_Toc168325083)

[Figure S16. The NOESY spectrum of compound **4** in CDCl_3_. 11](#_Toc168325084)

[Figure S17. The IR spectrum of compound **4**. 12](#_Toc168325085)

[Figure S18. The. UV spectrum of compound **4**. 12](#_Toc168325086)

[Figure S19. The HRESIMS spectrum of compound **5**. 13](#_Toc168325087)

[Figure S20. The ^1^H NMR (400MHz) spectrum of compound **5** in CD_3_OD. 13](#_Toc168325088)

[Figure S21. The ^13^C NMR (100MHz) spectrum of compound **5** in CD_3_OD. 14](#_Toc168325089)

[Figure S22. The HSQC spectrum of compound **5** in CD_3_OD. 14](#_Toc168325090)

[Figure S23. The ^1^H-^1^H COSY spectrum of compound **5** in CD_3_OD. 15](#_Toc168325091)

[Figure S24. The HMBC spectrum of compound **5** in CD_3_OD. 15](#_Toc168325092)

[Figure S25. The NOESY spectrum of compound **5** in CD_3_OD. 16](#_Toc168325093)

[Figure S26. The IR spectrum of compound **5**. 16](#_Toc168325094)

[Figure S27. The UV spectrum of compound **5**. 17](#_Toc168325095)

[Figure S28. The HRESIMS of compound **6**. 17](#_Toc168325096)

[Figure S29. The ^1^H NMR (400MHz) spectrum of compound **6** in CD_3_OD 18](#_Toc168325097)

[Figure S30. The ^13^C NMR (100MHz) spectrum of compound **6** in CD_3_OD. 18](#_Toc168325098)

[Figure S31. The HSQC spectrum of compound **6** in CD_3_OD. 19](#_Toc168325099)

[Figure S32. The ^1^H-^1^H COSY spectrum of compound **6** in CD_3_OD. 19](#_Toc168325100)

[Figure S33. The HMBC spectrum of compound **6** in CD_3_OD. 20](#_Toc168325101)

[Figure S34. The NOESY spectrum of compound **6** in CD_3_OD. 20](#_Toc168325102)

[Figure S35. The IR spectrum of compound **6**. 21](#_Toc168325103)

[Figure S36. The. UV spectrum of compound **6**. 21](#_Toc168325104)

[Figure S37. ^1^H NMR (400MHz) spectrum of (*S*)-MTPA esters **5** in pyridine-*d*_5_. 22](#_Toc168325105)

[Figure S38. The ^1^H-^1^H COSY spectrum of (*S*)-MTPA esters **5** in pyridine-*d*_5_. 22](#_Toc168325106)

[Figure S39. ^1^H NMR (400MHz) spectrum of (*R*)-MTPA esters **5** in pyridine-*d*_5_. 23](#_Toc168325107)

[Figure S40. The ^1^H-^1^H COSY spectrum of (*R*)-MTPA esters **5** in pyridine-*d*_5_. 23](#_Toc168325108)

[Figure S41. ^1^H NMR (400MHz) spectrum of (*S*)-MTPA esters **6** in pyridine-*d*_5_. 24](#_Toc168325109)

[Figure S42. The ^1^H-^1^H COSY spectrum of (*S*)-MTPA esters **6** in pyridine-*d_5_*. 24](#_Toc168325110)

[Figure S43. ^1^H NMR (400MHz) spectrum of (*R*)-MTPA esters **6** in pyridine-*d*_5_. 25](#_Toc168325111)

[Figure S44. The ^1^H-^1^H COSY spectrum of (*R*)-MTPA esters **6** in pyridine-*d*_5_. 25](#_Toc168325112)

[Table S1. The energy and Boltzmann distribution of the optimized conformers of (4*S*)-**4**. 26](#_Toc168325113)

[Table S2. Cartesian coordinates of the low-energy reoptimized conformers of (4*S*)-**4**. 26](#_Toc168325114)

**Crystal data for 1.** C_16_H_18_O_6_ (Mr = 306.30 g/mol), monoclinic, space group *P*2_1_, *a* = 8.2259(3) Å, *b* = 8.4033(2) Å, *c* = 23.3875(7) Å, *α* = 90◦, *β* = 98.174(3)◦, *γ* = 90◦, V = 1600.23(9)Å^3^, *Z* = 4, *T* = 293(2) K, *µ*(Cu Kα) = 0.818 mm^−1^, *D*_calc_ = 1.271 g/cm^3^, Independent reflections: 5850 (*R*_int_ = 0.471, *R*_sigma_ = 0.0373), *R*_1_ = 0.0708 (I > = 2σ (I)), *wR*_2_ = 0.2176. The goodness of fit was 1.092. Flack parameter = 0.15 (11), Hooft parameter = 0.12 (10).

| SPECTRUM-simulation: |  |  |  |  |  |
| --- | --- | --- | --- | --- | --- |
| *m/z* | Species | Theo. Mass | Delta (ppm) | DBE | Composition |
| 307.1179 | [M+H]^+^ | 307.1176 | 0.86 | 8 | C_16_H_19_O_6_ |
| Figure S1. The HR-ESIMS of compound 1. | | | | | |

|  |
| --- |
| Figure S2. The ^1^H NMR (400 MHz) spectrum of compound 1 in CDCl_3_. |

|  |
| --- |
| Figure S3. The ^13^C NMR (100 MHz) spectrum of compound 1 in CDCl_3_. |

|  |
| --- |
| Figure S4. The HSQC spectrum of compound 1 in CDCl_3_. |

|  |
| --- |
| Figure S5. The ^1^H-^1^H COSY spectrum of compound 1 in CDCl_3_. |

|  |
| --- |
| Figure S6. The HMBC spectrum of compound 1 in CDCl_3_. |

|  |
| --- |
| Figure S7. The NOESY spectrum of compound 1 in CDCl_3_. |

| 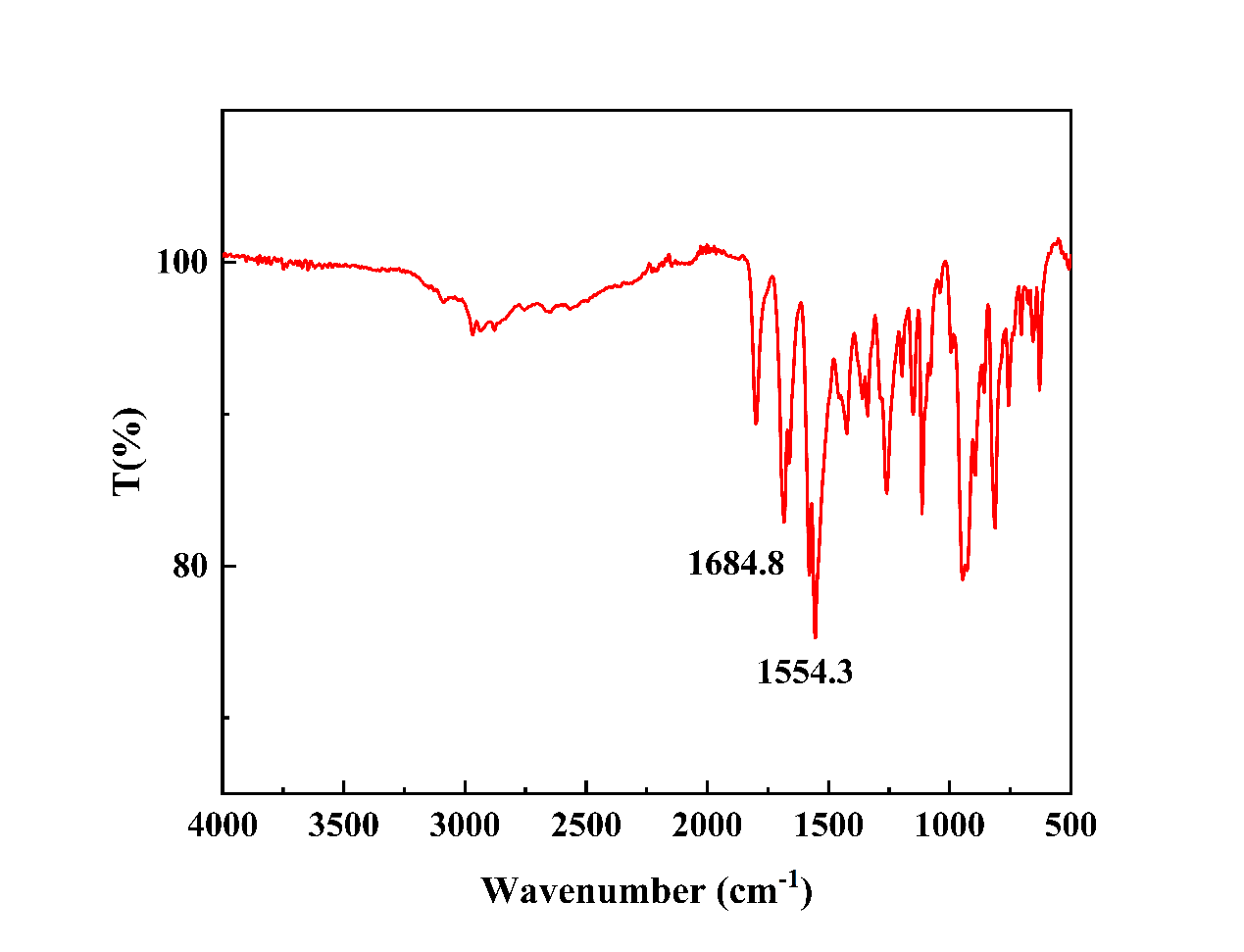 |
| --- |
| Figure S8. The IR spectrum of compound 1. |

| 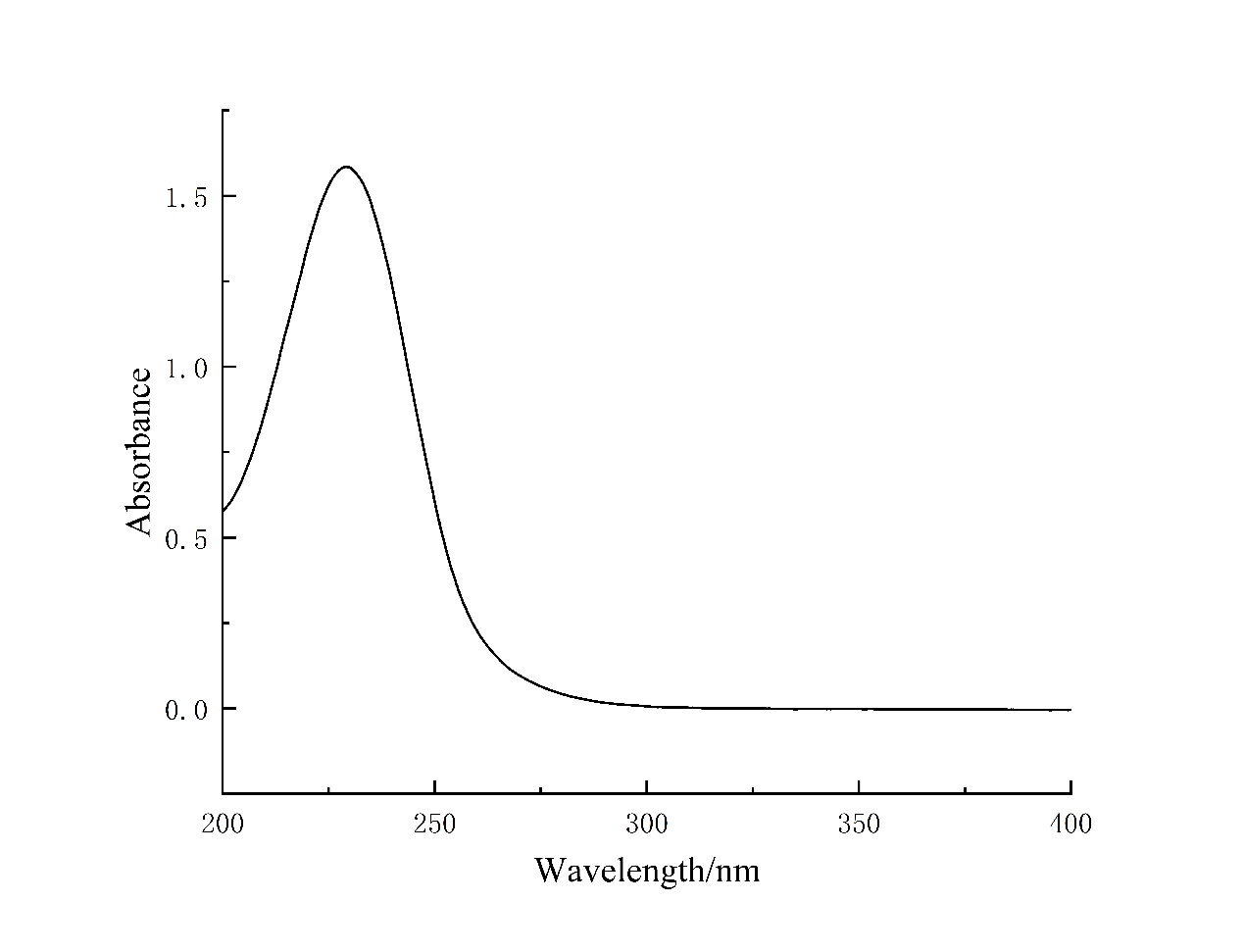 |
| --- |
| Figure S9. The. UV spectrum of compound 1. |

|  | | | | | | |
| --- | --- | --- | --- | --- | --- | --- |
| SPECTRUM-simulation: |  |  |  |  |  |  |
| *m/z* | Species | Theo. Mass | Delta (ppm) | DBE | Composition |  |
| 255.1230 | [M+H]^+^ | 255.1227 | 0.28 | 5 | C_13_H_19_O_5_ |  |
| Figure S10. The HR-ESIMS of compound 4 . | | | | | |  |

|  |
| --- |
| Figure S11. The ^1^H NMR (400 MHz) spectrum of compound 4 in CDCl_3_. |

|  |
| --- |
| Figure S12. The ^13^C NMR (100 MHz) spectrum of compound 4 in CDCl_3_. |

|  |
| --- |
| Figure S13. The HSQC spectrum of compound 4 in CDCl_3_. |

|  |
| --- |
| Figure S14. The ^1^H-^1^H COSY spectrum of compound 4 in CDCl_3_. |

|  |
| --- |
| Figure S15. The HMBC spectrum of compound 4 in CDCl_3_. |

|  |
| --- |
| Figure S16. The NOESY spectrum of compound 4 in CDCl_3_. |

| 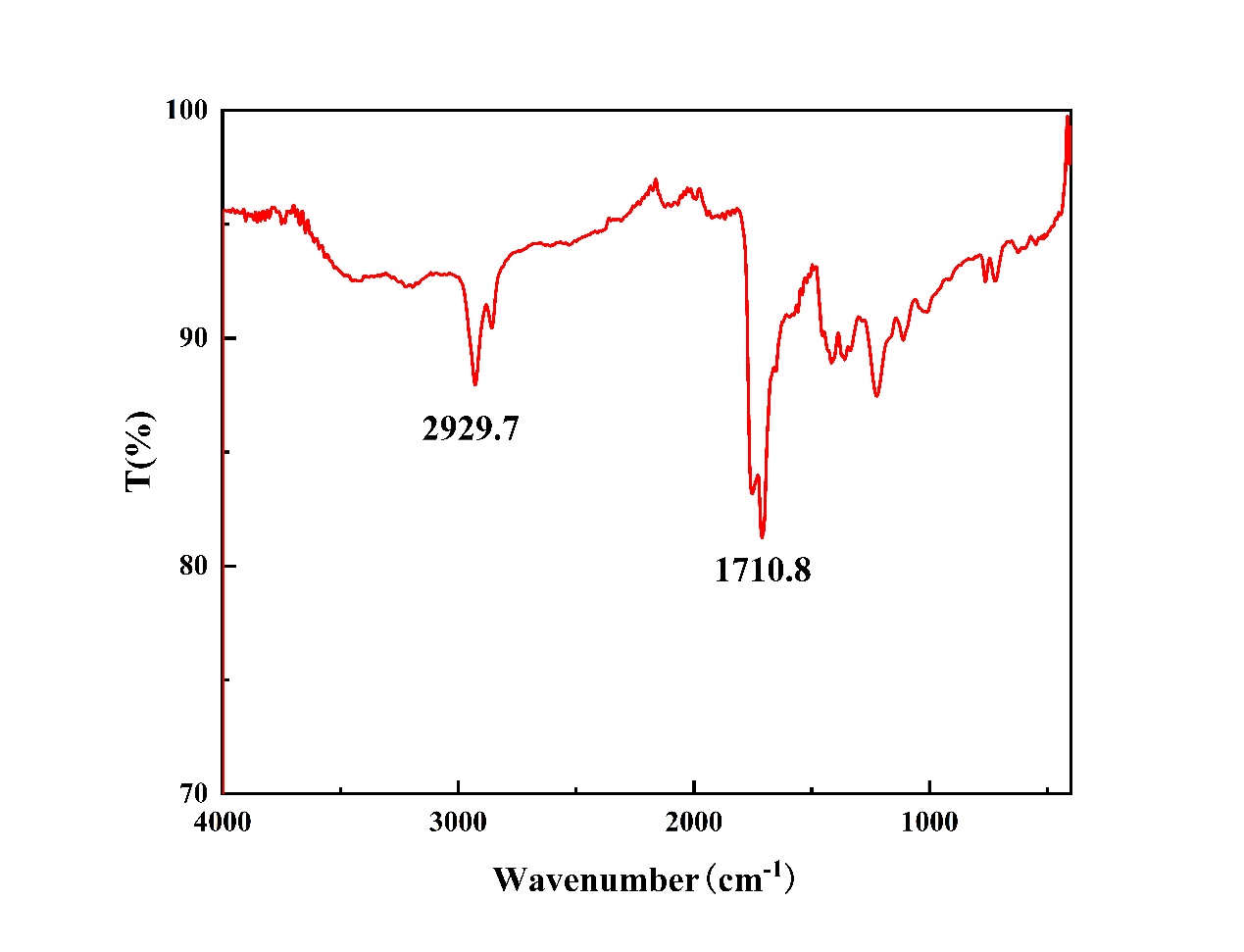 |
| --- |
| Figure S17. The IR spectrum of compound 4. |

| 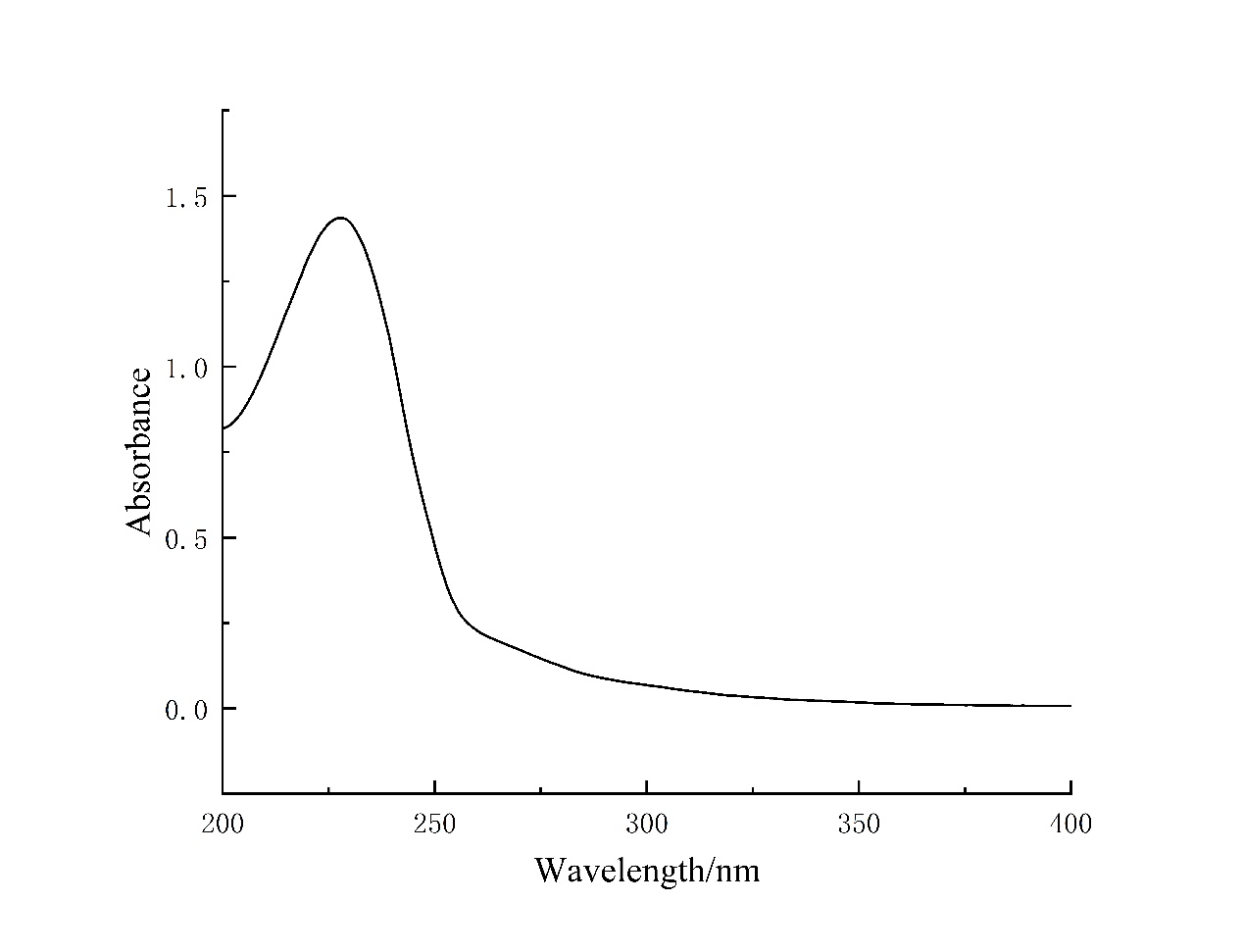 |
| --- |
| Figure S18. The. UV spectrum of compound 4. |

|  | | | | | |
| --- | --- | --- | --- | --- | --- |
| SPECTRUM-simulation: |  |  |  |  |  |
| m/z | Species | Theo. Mass | Delta (ppm) | RDB | Composition |
| 271.1177 | [M-H]^-^ | 271.1182 | 0.130 | 4.5 | C_13_H_19_O_6_ |
| Figure S19. The HRESIMS spectrum of compound 5. | | | | | |

|  |
| --- |
| Figure S20. The ^1^H NMR (400MHz) spectrum of compound 5 in CD_3_OD. |

|  |
| --- |
| Figure S21. The ^13^C NMR (100MHz) spectrum of compound 5 in CD_3_OD. |

|  |
| --- |
| Figure S22. The HSQC spectrum of compound 5 in CD_3_OD. |

|  |
| --- |
| Figure S23. The ^1^H-^1^H COSY spectrum of compound 5 in CD_3_OD. |

|  |
| --- |
| Figure S24. The HMBC spectrum of compound 5 in CD_3_OD. |

|  |
| --- |
| Figure S25. The NOESY spectrum of compound 5 in CD_3_OD. |

| 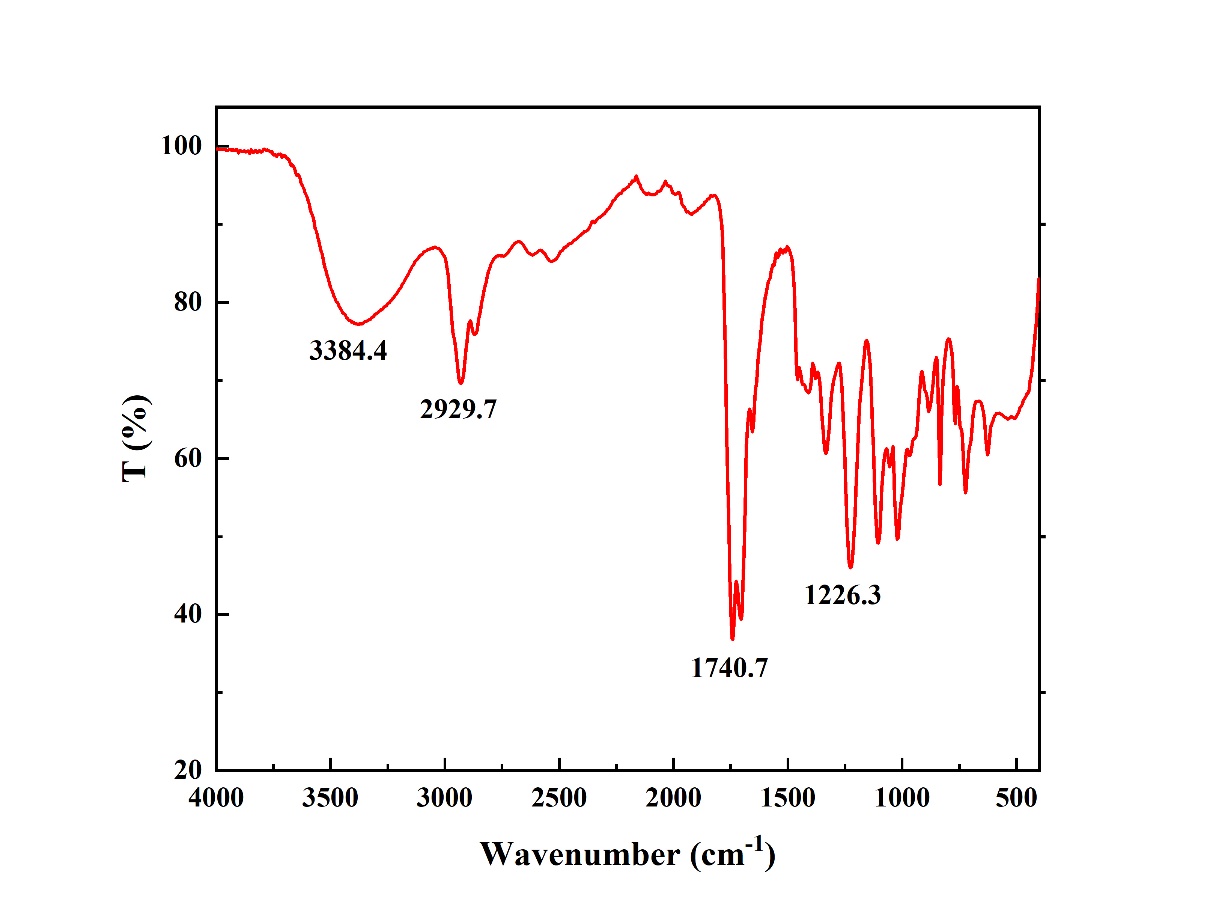 |
| --- |
| Figure S26. The IR spectrum of compound 5. |

| 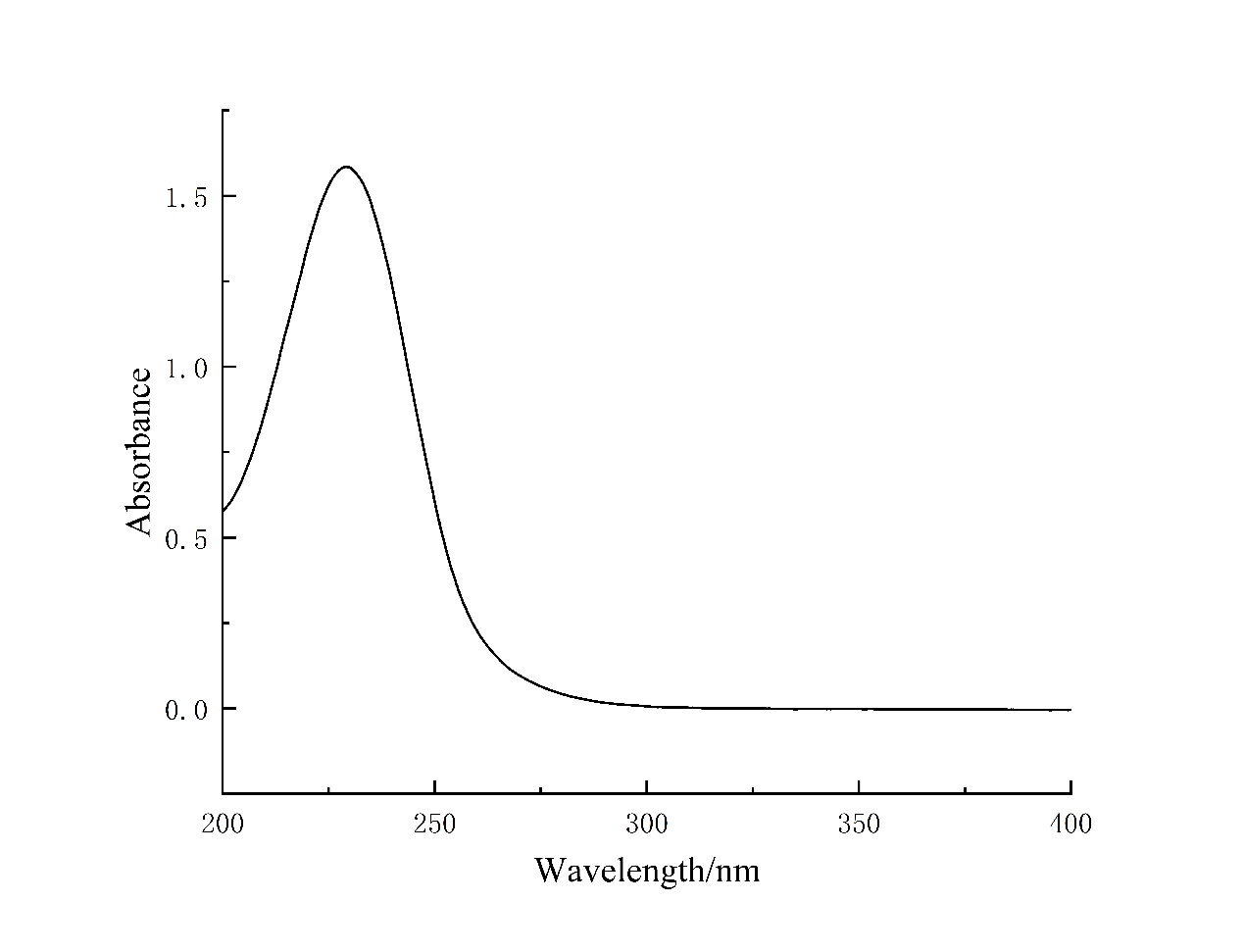 |
| --- |
| Figure S27. The UV spectrum of compound 5. |

|  | | | | | |
| --- | --- | --- | --- | --- | --- |
| SPECTRUM-simulation: |  |  |  |  |  |
| m/z | Species | Theo. Mass | Delta (ppm) | DBE | Composition |
| 271.1179 | [M-H]^-^ | 271.1182 | 0.130 | 4.5 | C_13_H_19_O_6_ |
| Figure S28. The HRESIMS of compound 6. | | | | | |

|  |
| --- |
| Figure S29. The ^1^H NMR (400MHz) spectrum of compound 6 in CD_3_OD |

|  |
| --- |
| Figure S30. The ^13^C NMR (100MHz) spectrum of compound 6 in CD_3_OD. |

|  |
| --- |
| Figure S31. The HSQC spectrum of compound 6 in CD_3_OD. |

|  |
| --- |
| Figure S32. The ^1^H-^1^H COSY spectrum of compound 6 in CD_3_OD. |

|  |
| --- |
| Figure S33. The HMBC spectrum of compound 6 in CD_3_OD. |

|  |
| --- |
| Figure S34. The NOESY spectrum of compound 6 in CD_3_OD. |

| 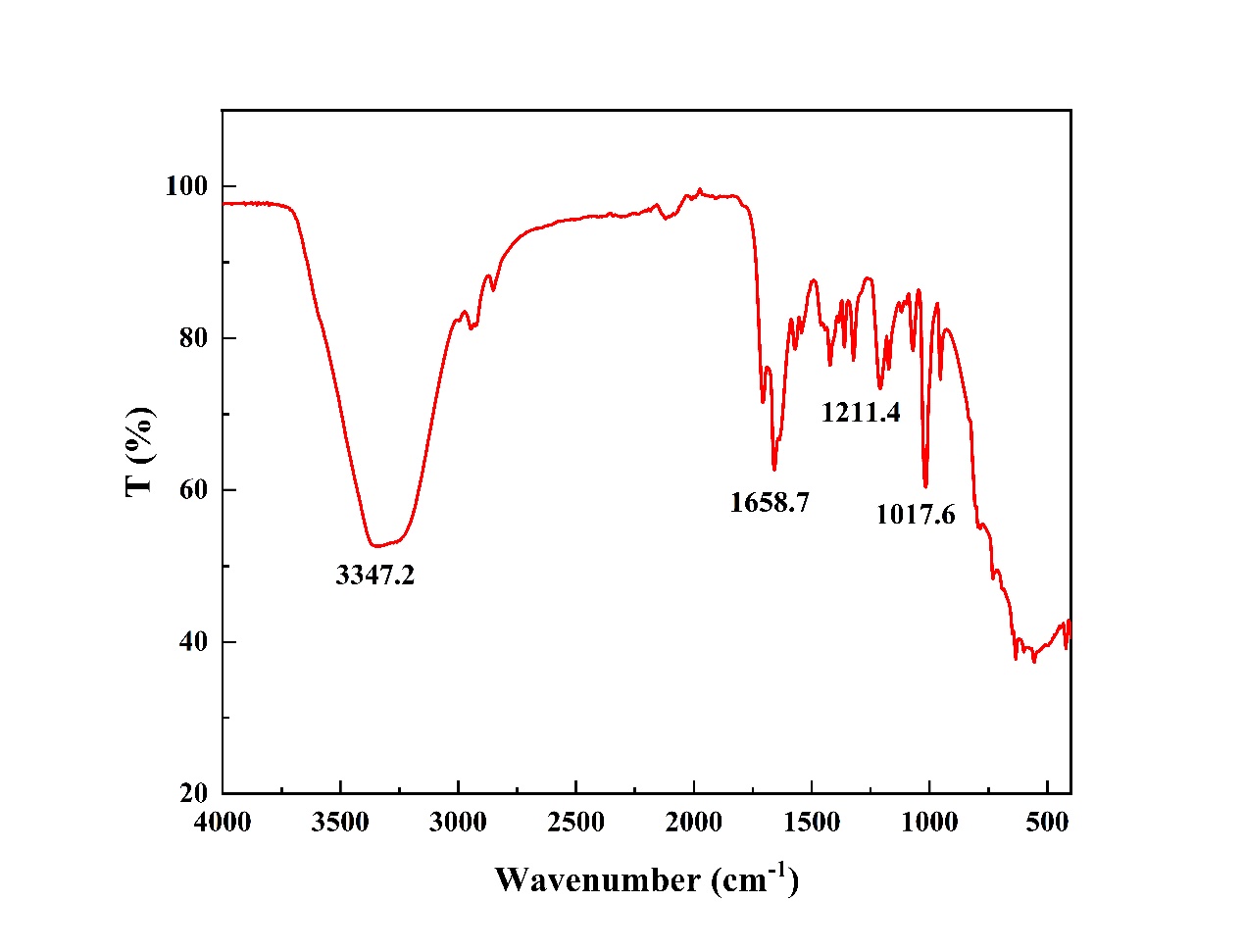 |
| --- |
| Figure S35. The IR spectrum of compound 6. |

| 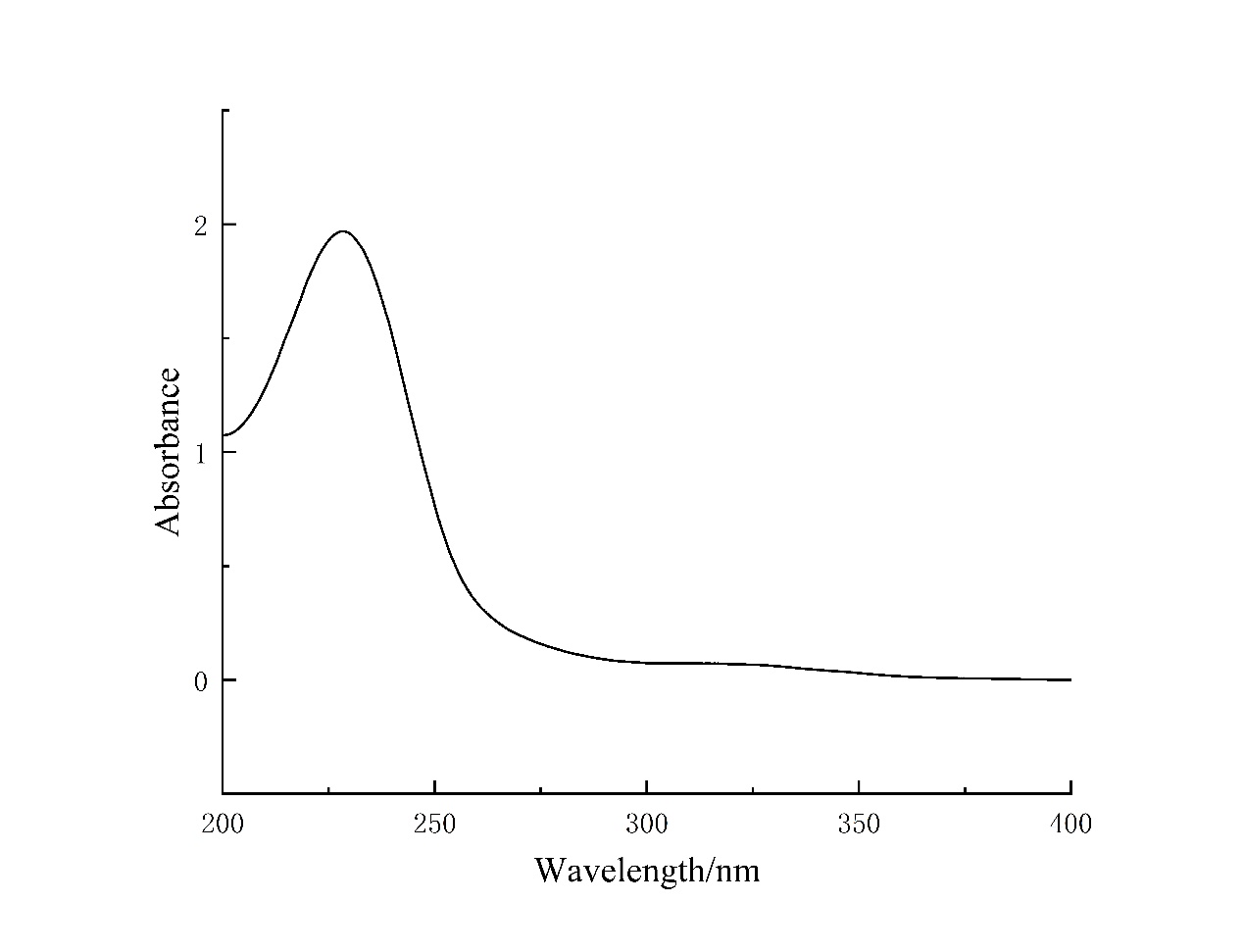 |
| --- |
| Figure S36. The. UV spectrum of compound 6. |

##### Figure S37. ^1^H NMR (400MHz) spectrum of (*S*)-MTPA esters 5 in pyridine-*d*_5_.

##### **Figure S38.** The ^1^H-^1^H COSY spectrum of (*S*)-MTPA esters 5 in pyridine-*d*_5_.

##### Figure S39. ^1^H NMR (400MHz) spectrum of (*R*)-MTPA esters 5 in pyridine-*d*_5_.

##### Figure S40. The ^1^H-^1^H COSY spectrum of (*R*)-MTPA esters 5 in pyridine-*d*_5_.

##### Figure S41. ^1^H NMR (400MHz) spectrum of (*S*)-MTPA esters 6 in pyridine-*d*_5_.

##### **Figure S42. The ^1^H-^1^H COSY spectrum of (*S*)-MTPA esters 6 in pyridine-*d_5_*.**

##### Figure S43. ^1^H NMR (400MHz) spectrum of (*R*)-MTPA esters 6 in pyridine-*d*_5_.

##### Figure S44. The ^1^H-^1^H COSY spectrum of (*R*)-MTPA esters 6 in pyridine-*d*_5_.

##### Table S1. The energy and Boltzmann distribution of the optimized conformers of (4*S*)-4.

|  | Conformers | Relative energies (kcal/mol) | Boltzmann distribution (%) |
| --- | --- | --- | --- |
| (4*S*)-**4** | **a** | 0 | 42.28% |
|  | **b** | 0.667042566 | 13.70% |
|  | **c** | 1.20544569 | 5.52% |
|  | **d** | 0.180095218 | 31.19% |
|  | **e** | 2.154240007 | 1.11% |
|  | **f** | 1.137674667 | 6.19% |

##### Table S2. Cartesian coordinates of the low-energy reoptimized conformers of (4*S*)-4.

| Conformer **a** | | | | Conformer **b** | | | |
| --- | --- | --- | --- | --- | --- | --- | --- |
| 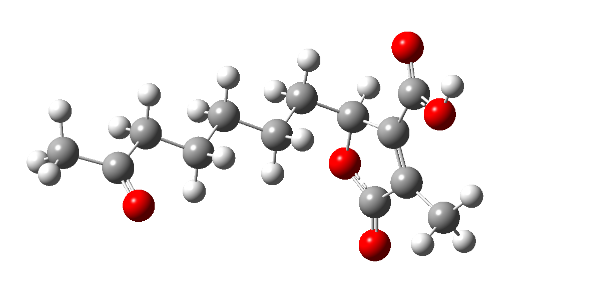 | | | | 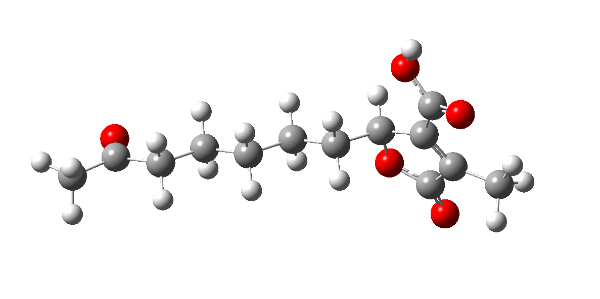 | | | |
| Atom | X axis  (Å) | Y axis  (Å) | Z axis  (Å) | Atom | X axis  (Å) | Y axis  (Å) | Z axis  (Å) |
| C | 2.099715 | -0.02432 | -1.385175 | C | 1.656485 | -0.091246 | -0.676456 |
| C | 2.620415 | 0.410085 | -0.04303 | C | 2.970696 | 0.381966 | -0.117754 |
| C | 2.981572 | -0.649314 | 0.683577 | C | 3.71587 | -0.657937 | 0.260147 |
| C | 2.710816 | -1.852092 | -0.157197 | C | 2.932884 | -1.884778 | -0.060917 |
| O | 2.214435 | -1.456895 | -1.340956 | O | 1.763291 | -1.525013 | -0.615182 |
| C | 3.552323 | -0.806186 | 2.043782 | C | 5.06372 | -0.762468 | 0.868457 |
| O | 2.892259 | -3.00792 | 0.118296 | O | 3.242998 | -3.032704 | 0.114986 |
| C | 2.716301 | 1.851265 | 0.267775 | C | 3.354452 | 1.809564 | -0.051689 |
| O | 2.88213 | 2.111783 | 1.56416 | O | 2.6091 | 2.556322 | -0.872193 |
| O | 2.645782 | 2.708712 | -0.57901 | O | 4.231105 | 2.259946 | 0.641864 |
| C | 0.662155 | 0.372466 | -1.695251 | C | 0.437897 | 0.362489 | 0.113651 |
| C | -0.349486 | -0.023546 | -0.626369 | C | -0.878842 | -0.124399 | -0.479793 |
| C | -1.779166 | 0.312283 | -1.035983 | C | -2.081695 | 0.322882 | 0.34326 |
| C | -2.806102 | -0.089183 | 0.017066 | C | -3.409426 | -0.126907 | -0.256269 |
| C | -4.225566 | 0.249066 | -0.40742 | C | -4.598506 | 0.319566 | 0.577887 |
| C | -5.298229 | -0.11861 | 0.591016 | C | -5.949796 | -0.065403 | 0.022763 |
| C | -6.720815 | 0.047694 | 0.122299 | C | -7.137427 | 0.243107 | 0.898135 |
| O | -5.037512 | -0.531429 | 1.701175 | O | -6.078896 | -0.60123 | -1.057572 |
| H | 2.75958 | 0.326193 | -2.181336 | H | 1.554982 | 0.177819 | -1.730396 |
| H | 3.957632 | 0.126565 | 2.423952 | H | 5.544633 | 0.208202 | 0.944217 |
| H | 2.774189 | -1.150786 | 2.729594 | H | 4.979455 | -1.19521 | 1.868113 |
| H | 4.329699 | -1.570826 | 2.0299 | H | 5.683551 | -1.439652 | 0.278169 |
| H | 2.955583 | 3.069161 | 1.676562 | H | 2.892453 | 3.477326 | -0.797871 |
| H | 0.396709 | -0.084756 | -2.65154 | H | 0.444799 | 1.454374 | 0.144497 |
| H | 0.649393 | 1.454138 | -1.846604 | H | 0.547737 | 0.008825 | 1.143288 |
| H | -0.1172 | 0.484867 | 0.314742 | H | -0.87025 | -1.215571 | -0.548302 |
| H | -0.275072 | -1.097206 | -0.428316 | H | -0.975697 | 0.25566 | -1.50224 |
| H | -2.010122 | -0.190959 | -1.981041 | H | -2.07421 | 1.41475 | 0.432948 |
| H | -1.857495 | 1.387386 | -1.23055 | H | -1.987581 | -0.071663 | 1.360841 |
| H | -2.579631 | 0.411679 | 0.961869 | H | -3.419564 | -1.216316 | -0.351734 |
| H | -2.729789 | -1.162796 | 0.211063 | H | -3.508485 | 0.271941 | -1.269237 |
| H | -4.475737 | -0.232852 | -1.358768 | H | -4.602154 | 1.409963 | 0.693158 |
| H | -4.32713 | 1.324268 | -0.596873 | H | -4.533864 | -0.074146 | 1.598321 |
| H | -6.850964 | 1.001045 | -0.392279 | H | -7.036199 | 1.22271 | 1.367012 |
| H | -6.947759 | -0.741284 | -0.599578 | H | -8.060456 | 0.191412 | 0.324038 |
| H | -7.412253 | -0.025501 | 0.959336 | H | -7.177795 | -0.497616 | 1.701419 |
| Conformer **c** | | | | Conformer **d** | | | |
| 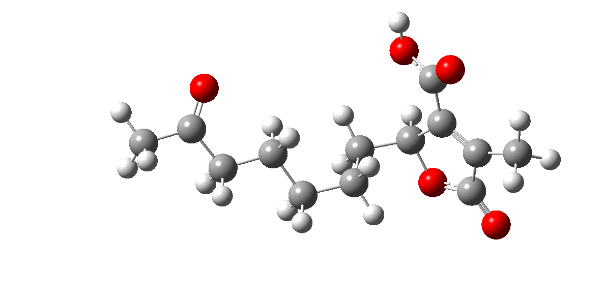 | | | | 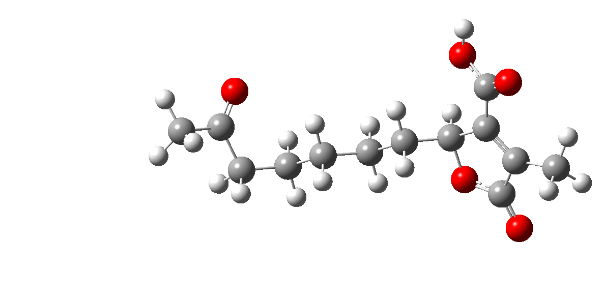 | | | |
| Atom | X axis  (Å) | Y axis  (Å) | Z axis  (Å) | Atom | X axis  (Å) | Y axis  (Å) | Z axis  (Å) |
| C | -1.735695 | -0.333546 | 1.240531 | C | 1.531573 | -0.20884 | -0.695183 |
| C | -2.297114 | 0.636421 | 0.236033 | C | 2.710742 | 0.518701 | -0.111028 |
| C | -3.269679 | 0.061378 | -0.472776 | C | 3.634712 | -0.349302 | 0.304022 |
| C | -3.409959 | -1.329709 | 0.043297 | C | 3.112543 | -1.710325 | -0.005322 |
| O | -2.517125 | -1.522672 | 1.028673 | O | 1.908648 | -1.593875 | -0.590298 |
| C | -4.141052 | 0.553679 | -1.566211 | C | 4.956222 | -0.169194 | 0.951889 |
| O | -4.176478 | -2.183066 | -0.316138 | O | 3.63479 | -2.773342 | 0.2 |
| C | -1.817019 | 2.029558 | 0.103443 | C | 2.81144 | 1.993079 | -0.052666 |
| O | -1.13842 | 2.420283 | 1.186316 | O | 1.96174 | 2.579627 | -0.901225 |
| O | -2.005538 | 2.738741 | -0.85263 | O | 3.56595 | 2.60508 | 0.660673 |
| C | -0.25277 | -0.648031 | 1.089704 | C | 0.217954 | 0.021827 | 0.036305 |
| C | 0.15834 | -1.098264 | -0.30901 | C | -0.950785 | -0.741207 | -0.575812 |
| C | 1.622201 | -1.527562 | -0.391117 | C | -2.259549 | -0.479804 | 0.160635 |
| C | 2.613151 | -0.388109 | -0.167806 | C | -3.436201 | -1.243121 | -0.439713 |
| C | 4.054421 | -0.846565 | -0.314398 | C | -4.744381 | -1.005681 | 0.301164 |
| C | 5.090643 | 0.235246 | -0.114562 | C | -5.34461 | 0.372028 | 0.118217 |
| C | 6.516044 | -0.148869 | -0.417246 | C | -6.39052 | 0.775725 | 1.123157 |
| O | 4.798223 | 1.351437 | 0.259262 | O | -5.015647 | 1.106692 | -0.788971 |
| H | -1.93939 | 0.003822 | 2.258695 | H | 1.420264 | 0.012298 | -1.759481 |
| H | -4.02543 | 1.622797 | -1.717515 | H | 5.664863 | -0.900327 | 0.561858 |
| H | -3.887309 | 0.038903 | -2.496082 | H | 5.338687 | 0.837522 | 0.805825 |
| H | -5.183295 | 0.318942 | -1.344235 | H | 4.863593 | -0.346426 | 2.026415 |
| H | -0.836419 | 3.328804 | 1.053715 | H | 2.057626 | 3.53827 | -0.824616 |
| H | -0.003492 | -1.422551 | 1.819353 | H | 0.009281 | 1.093927 | 0.013544 |
| H | 0.292056 | 0.250024 | 1.384577 | H | 0.352492 | -0.26011 | 1.084902 |
| H | -0.018974 | -0.292579 | -1.028748 | H | -0.734989 | -1.813096 | -0.564991 |
| H | -0.47308 | -1.935791 | -0.617305 | H | -1.06078 | -0.452228 | -1.626352 |
| H | 1.802598 | -1.967624 | -1.376357 | H | -2.472891 | 0.593267 | 0.147009 |
| H | 1.807102 | -2.321997 | 0.340317 | H | -2.144721 | -0.763276 | 1.212643 |
| H | 2.478249 | 0.045702 | 0.825259 | H | -3.216138 | -2.313991 | -0.422224 |
| H | 2.410376 | 0.413069 | -0.884692 | H | -3.559061 | -0.963026 | -1.488903 |
| H | 4.22677 | -1.297034 | -1.297885 | H | -4.630271 | -1.196555 | 1.372574 |
| H | 4.282067 | -1.640141 | 0.407164 | H | -5.513379 | -1.709107 | -0.039244 |
| H | 6.756734 | -1.118289 | 0.022133 | H | -6.904412 | 1.679612 | 0.802576 |
| H | 6.633135 | -0.248628 | -1.499522 | H | -7.107842 | -0.032145 | 1.279561 |
| H | 7.203946 | 0.611026 | -0.052039 | H | -5.901005 | 0.958614 | 2.083482 |
| Conformer **e** | | | | Conformer **f** | | | |
| 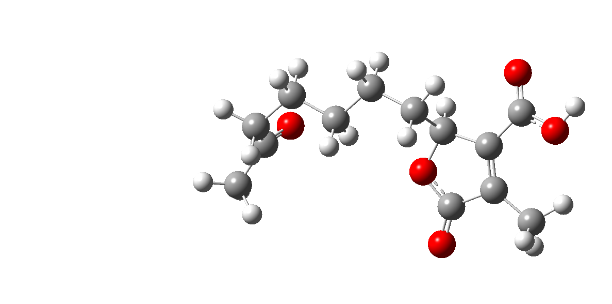 | | | | 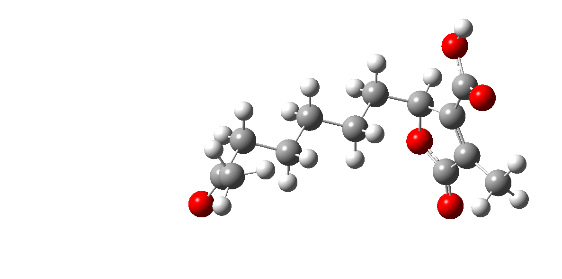 | | | |
| Atom | X axis  (Å) | Y axis  (Å) | Z axis  (Å) | Atom | X axis  (Å) | Y axis  (Å) | Z axis  (Å) |
| C | 1.121618 | -0.400575 | 0.323104 | C | 2.115539 | -0.141385 | -1.327673 |
| C | 2.579291 | -0.05587 | 0.181434 | C | 2.578484 | 0.390742 | 0.001599 |
| C | 2.731137 | 1.243603 | -0.079493 | C | 2.887566 | -0.613023 | 0.823674 |
| C | 1.361072 | 1.831347 | -0.112531 | C | 2.655391 | -1.874211 | 0.064211 |
| O | 0.463315 | 0.866155 | 0.141145 | O | 2.223168 | -1.567415 | -1.17063 |
| C | 3.918716 | 2.102679 | -0.310467 | C | 3.377807 | -0.648953 | 2.222275 |
| O | 1.046926 | 2.973559 | -0.316935 | O | 2.81608 | -3.006603 | 0.433765 |
| C | 3.60574 | -1.096217 | 0.402525 | C | 2.683126 | 1.834108 | 0.305843 |
| O | 4.801287 | -0.782899 | -0.09561 | O | 2.687273 | 2.573794 | -0.807197 |
| O | 3.376235 | -2.130795 | 0.980535 | O | 2.759315 | 2.303078 | 1.413591 |
| C | 0.623999 | -1.418808 | -0.696072 | C | 0.692164 | 0.229241 | -1.726333 |
| C | -0.781013 | -1.960995 | -0.440678 | C | -0.367073 | -0.106594 | -0.683007 |
| C | -1.911547 | -0.950225 | -0.605122 | C | -1.772069 | 0.244297 | -1.160197 |
| C | -3.288221 | -1.593448 | -0.464332 | C | -2.845798 | -0.123591 | -0.142759 |
| C | -4.427232 | -0.601987 | -0.654243 | C | -4.254412 | 0.246376 | -0.627869 |
| C | -4.597365 | 0.404784 | 0.463299 | C | -5.294309 | -0.147061 | 0.395349 |
| C | -5.404407 | 1.631081 | 0.128762 | C | -5.584399 | 0.850097 | 1.48466 |
| O | -4.125433 | 0.23382 | 1.567299 | O | -5.853534 | -1.223055 | 0.34589 |
| H | 0.900123 | -0.736121 | 1.339376 | H | 2.808395 | 0.146334 | -2.120758 |
| H | 4.827926 | 1.645451 | 0.068466 | H | 2.561467 | -0.938638 | 2.888858 |
| H | 4.042419 | 2.276908 | -1.382214 | H | 4.158488 | -1.404002 | 2.321842 |
| H | 3.767186 | 3.074405 | 0.160155 | H | 3.752057 | 0.320395 | 2.539586 |
| H | 5.417139 | -1.49817 | 0.113639 | H | 2.749763 | 3.505939 | -0.559674 |
| H | 1.326259 | -2.254378 | -0.679884 | H | 0.470852 | -0.284286 | -2.66497 |
| H | 0.689095 | -0.964462 | -1.689353 | H | 0.682542 | 1.299819 | -1.942357 |
| H | -0.820533 | -2.394729 | 0.564278 | H | -0.15989 | 0.430415 | 0.247929 |
| H | -0.946145 | -2.788437 | -1.137273 | H | -0.323581 | -1.173704 | -0.445389 |
| H | -1.830855 | -0.477947 | -1.590501 | H | -1.975852 | -0.272637 | -2.103566 |
| H | -1.807174 | -0.151596 | 0.130999 | H | -1.826808 | 1.316827 | -1.373866 |
| H | -3.376209 | -2.062353 | 0.519154 | H | -2.63548 | 0.382095 | 0.804591 |
| H | -3.392963 | -2.390504 | -1.205748 | H | -2.805611 | -1.198501 | 0.055934 |
| H | -5.388592 | -1.124697 | -0.724636 | H | -4.471024 | -0.276432 | -1.561091 |
| H | -4.32263 | -0.060432 | -1.599299 | H | -4.304047 | 1.322947 | -0.809627 |
| H | -5.677316 | 2.171214 | 1.03314 | H | -6.135183 | 1.689156 | 1.050924 |
| H | -4.799329 | 2.280288 | -0.510053 | H | -6.178844 | 0.398327 | 2.275998 |
| H | -6.297763 | 1.364632 | -0.438854 | H | -4.657761 | 1.257305 | 1.893634 |
